# Could pooled samples method affect SARS-CoV-2 diagnosis accuracy using BGI and Sansure-Biotech RT-PCR kits used in Gabon, Central Africa?

**DOI:** 10.1371/journal.pone.0262733

**Published:** 2022-01-21

**Authors:** Rodrigue Mintsa-Nguema, Samira Zoa-Assoumou, Ludovic Mewono, Noé P. M’Bondoukwé, Paulin Essono, Krystina Mengue-Me-Ngou-Milama, Marlaine Boukandou-Mounanga, Jacques M. Ndong-Ngomo, Armel Mintsa-Ndong, Edgard B. Ngoungou, Marielle K. Bouyou-Akotet, Elvyre Mbongo-Kama

**Affiliations:** 1 Research Institute in Tropical Ecology, National Center for Scientific and Technological Research, Libreville, Gabon; 2 Professor Daniel Gahouma Laboratory, Ministry of Health, Libreville, Gabon; 3 Department of Bacteriology-Virology, University of Health Science, Libreville, Gabon; 4 Department of Biology, Higher Normal School, Libreville, Gabon; 5 Department of Parasitology-Mycology, University of Health Science, Libreville, Gabon; 6 National Laboratory of Public Health, Libreville, Gabon; 7 Institute of Pharmacopeia and Traditional Medicine, National Center for Scientific and Technological Research, Libreville, Gabon; Defense Threat Reduction Agency, UNITED STATES

## Abstract

This study aims at establishing specimens pooling approach for the detection of SARS-CoV-2 using the RT-PCR BGI and Sansure-Biotech kits used in Gabon. To validate this approach, 14 positive samples, stored at -20°C for three to five weeks were analyzed individually (as gold standard) and in pools of five, eight and ten in the same plate. We created 14 pools of 5, 8 and 10 samples using 40 μL from each of the selected positive samples mixed with 4, 7 and 9 confirmed negative counterparts in a total volume of 200 μL, 320 μL and 400 μL for the pools of 5, 8 and 10 respectively. Both individual and pooled samples testing was conducted according to the BGI and Sansure-Biotech RT-PCR protocols used at the Professor Daniel Gahouma Laboratory (PDGL). Furthermore, the pooling method was also tested by comparing results of 470 unselected samples tested in 94 pools and individually. Results of our experiment showed that using a BGI single positive sample with cycle threshold (Ct) value of 28.42, confirmed by individual testing, detection occurred in all the pools. On the contrary samples with Ct >31 were not detected in pools of 10 and for these samples (Ct value as high as 37.17) their detection was possible in pool of 8. Regarding the Sansure-Biotech kit, positive samples were detected in all the pool sizes tested, irrespective of their Ct values. The specificity of the pooling method was 100% for the BGI and Sansure-Biotech RT-PCR assays. The present study found an increase in the Ct values with pool size for the BGI and Sansure-Biotech assays. This trend was statistically significant (Pearson’s r = 0.978; *p* = 0,022) using the BGI method where the mean Ct values were 24.04±1.1, 26.74±1.3, 27.91±1.1 and 28.32±1.1 for the individual, pool of 5, 8 and 10 respectively. The testing of the 470 samples showed that one of the 94 pools had a positive test similar to the individual test using the BGI and Sansure-Biotech kits. The saving of time and economizing test reagents by using the pooling method were demonstrated in this study. Ultimately, the pooling method could be used for the diagnosis of SARS-CoV-2 without modifying the accuracy of results in Gabon. We recommend a maximum pool size of 8 for the BGI kit. For the Sansure-Biotech kit, a maximum pool size of 10 can be used without affecting its accuracy compared to the individual testing.

## Introduction

The outbreak of COVID-19 caused by the novel coronavirus (SARS-CoV-2) has spread globally [1] since it was first reported in Wuhan, China, in December 2019 [2]. As in other countries Gabon is also affected by COVID-19 and according to the May 2021 report, 11,457 cases and 71 deaths have so far been reported after conducting 456,358 tests [3]. Since the report of the first case in Libreville, the capital city of Gabon, in March 2020, the country has conducted massive SARS-CoV-2 testing to identify infected individuals and to trace cases in order to mitigate its spread. To support this mitigation strategy, > 15 laboratories and > 60 testing centers were created in the country. To date, Gabon is among the 12 African countries that have performed more than 10 tests for 100,000 people [4]. Since July 2020, Gabon has increased its diagnostic capacity by conducting more than 2,000 tests per day, compared to the 200 tests per week at the start of the pandemic.

This mass SARS-CoV-2 screening strategy in Gabon involve additional burden in terms of laboratory equipment, qualified staff and reagents required for testing. The reverse-transcription polymerase chain reaction (RT-PCR) is recommended for the detection of the SARS-CoV-2 infection [5, 6]. This method is identified as one of the primary bottlenecks in the entire COVID-19 testing sphere [7]. Indeed, each sample tested requires laboratory resources that are increasingly in short supply as the number of PCR reactions performed globally increases tremendously. The risk of lack of test supplies is obvious, therefore, it is important to develop alternative testing strategies to conserve SARS-CoV-2 diagnostic resources and to increase the testing capacity. Sample pooling has been proposed to improve the testing capacity and resources for SARS-CoV-2 infection and other diseases [8, 9]. Before SARS-CoV-2, sample pooling was already used to test blood prior to transfusion for the detection of the human immunodeficiency virus and hepatitis B and C viruses [10]. Sample pooling is currently used globally for SARS-CoV-2 diagnosis without loss in diagnostic accuracy compared to individual testing when the disease prevalence is low [11–13].

Concerning the pooling procedure, the major element to guarantee its success depends on the laboratory’s protocol, especially the limit of detection of the RT-PCR kits used and the optimal pool size (number of samples in a pool) that depends on the prevalence of disease in the studied population [14, 15]. In Gabon, two main types of RT-PCR protocols are used for the detection of SARS-CoV-2. One requires a separate viral RNA extraction step prior to conducting the RT-PCR (BGI kit). The other is a single reaction assay using reagents to conduct nucleic acid extraction and RT-PCR amplification directly from the NP/OP swab samples (Sansure-Biotech kit). These two assays are effective for the diagnosis of SARS-CoV-2 in individual samples [16]. However, we do not know the optimized conditions for their use in the pooling method. The main objective of this study was to evaluate the diagnostic accuracy and efficacy of the pooling method using the BGI and the Sansure-Biotech kits for the detection of SARS-CoV-2 in order to suggest it for use in Gabon.

## Material and methods

### Study framework and ethical statement

This study was conducted as a part of the diagnostic procedure for the COVID-19 policy in Gabon at the Professor Daniel Gahouma Laboratory (PDGL). The PDGL was created in order to respond to the country’s challenges with the SARS-CoV-2 testing capacity. The PDGL uses the BGI and the Sansure-Biotech kits for SARS-CoV-2 testing. This laboratory has the capacity to perform 10,000 tests per day and manages >3/4 of tests performed in the Gabon. All samples used in this study were analyzed anonymously.

### BGI test for SARS-CoV-2 detection

The procedure for this test involves an automatic RNA extraction phase followed by the reverse transcription and amplification phases. Viral RNA was extracted from 180 μL of each sample pool using the MGISP-960 automatic extraction approach following manufacturer’s instructions (Wuhan MGI Tech Co., Ltd, China). The RT-PCR amplification was conducted using the 2019-nCoV real-time fluorescent RT-PCR kit (BGI, Shenzhen, China). 10 μL RNA was added to 20 μL PCR mix, giving a total reaction volume of 30 μL as indicated by the manufacturer. The 2019-nCoV *ORF1ab* was the target region for amplification. Amplification was conducted using the MA 6000 thermocycler (Suzhou Molarray Co., Ltd, China) with reverse transcription at 50°C for 20 min, a cDNA initial denaturation step at 95°C for 10 min and 40 cycles at 95°C for 15 s (cDNA denaturation) and 60°C for 30 s (annealing, elongation and collection fluorochrome). According to this method, a sample was declared positive for 2019-nCoV when the cycle threshold (Ct) value of the target *ORF1ab* region was ≤ 38 (≤ 32 for highly positive sample).

### Sansure-Biotech test for SARS-CoV-2 detection

This assay consists of a single reaction using the sample release reagent for RNA extraction and the 2019-nCoV-PCR master mix (2019-nCoV-PCR Mix + 2019-nCoV-PCR-Enzyme Mix) to conduct the RT-PCR amplification. Sansure-Biotech test uses the novel Coronavirus (2019-nCoV) nucleic acid diagnostic kit (PCR-fluorescence probe) and is a nucleic acid amplification test through reverse transcription polymerase chain reaction (RT-PCR). The test was carried out in a total volume of 50 μL including 10 μL of NP/OP swab sample, 10 μL of sample release reagent and 30 μL 2019-nCoV-PCR master mix. The novel coronavirus (2019-nCoV) *ORF1ab* and the conserved sequence of coding nucleocapsid protein *N* gene are the amplified target genes. The amplification was conducted using the MA 6000 thermocycler with the following conditions: a reverse transcription step at 50°C for 30 min, a cDNA initial denaturation step at 95°C for 1 min, 45 cycles at 95°C for 15s (denaturing) and 60°C for 30s (annealing, elongation and fluorescence collection) and a device cooling step at 25°C for 10s. For this method, positive 2019-nCoV samples were declared positive when cycle threshold (Ct) value of the target *ORF1ab* and/or *N* genes was ≤ 40.

### The pooling procedure

A one step sample pooling method from Shani-Narkiss et al [17] was used. Considering the prevalence of 0.025% for Gabon [3], pool sizes within the range of 5 and 10 were used to test the accuracy of the pool sample method against the individual sample test. The viral load was considered in the composition of the different pools.

**Fig 1:**
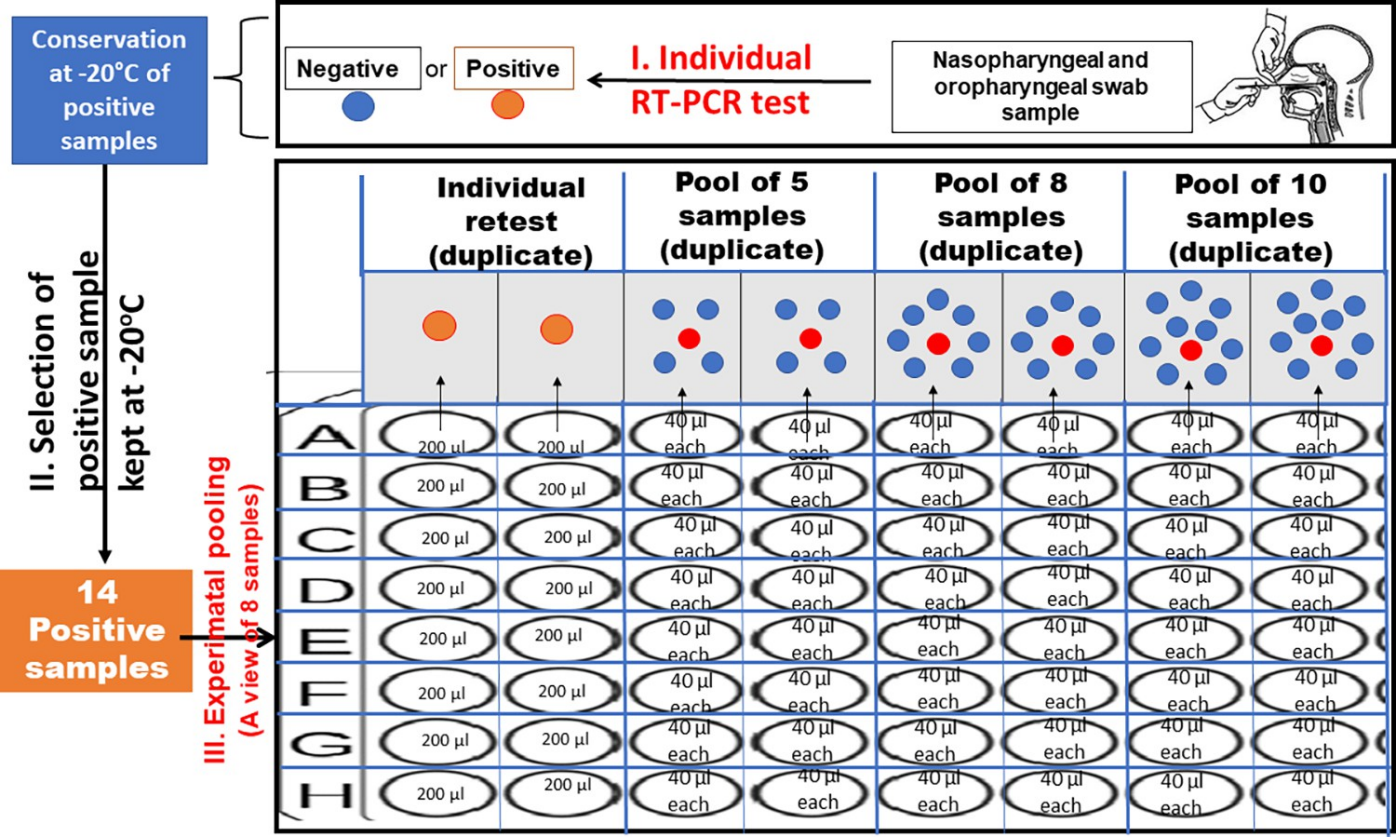
A display of the experimental pooling procedure in a deep-well plate. Here is a view for 8 positive samples. The rest of the 6 positive was placed in a different plate according the same organization method.

Samples were both nasopharyngeal and oropharyngeal swabs put together in a collection tube with a normal saline solution. Initially, we selected 14NP/OP positive swab samples that were kept at -20°C, some (5 samples) with high viral load (Ct values of *ORF1ab* and N genes < 32 respectively) and others (9 samples) with low viral load (32 ≤ Ct values of *ORF1ab* and N respectively < 40) as indicated by the Sansure-Biotech kit test results (**Table 1**). Each of these 14 samples was simultaneously retested individually and in pools of 5, 8 and 10 samples using the BGI and the Sansure-Biotech kits. Experimental pools were created by mixing 40 μL of each of the 14 samples with four, seven or nine other known negative samples (40 μL each) for the pools of 5, 8 and 10 respectively in a 96 deep-well plate as shown in **Fig 1**. For each pool of 5, 8 and 10, a total volume of 200 μL, 320 μL and 400 μL was reached respectively. The final volume of each pool was thoroughly mixed and the SARS-CoV-2 testing conducted according to the procedure of the kits used. Samples (individual and pooled) were placed in duplicates in a deep-well plate as shown in **Fig 1** for SARS-CoV-2 diagnosis. For each test (individual or pool), the outcome was considered positive if both duplicates are positive.

**Table 1:** Initial cycle threshold (Ct) values of the 14 samples used for the experimental pooling.

*Sample code*	Ct		Method
	*ORF1ab*	*N*	
200831P9400167*	26.37	24.86	Sansure-Biotech
200903P8600016*	26.09	22.44	
200902C2800011*	29.73	24.01	
200921P8600060*	25.50	23.59	
200923P0800072*	30.94	31.03	
200902P9400076	34.17	31.69	
200902P3300403	34.36	32.77	
200901P6500072	39.92	33.70	
200831P3300488	39.32	36.24	
200921P9400141	-	34.52	
200925P3300629	-	38.77	
200924C2500010	-	37.17	
200921P3300452	-	37.26	
200925P9501066	38.57	-	

*: samples with high viral load (Ct values of ORF1ab and N <32).

### Validation of the pooling method

The outcome of the test for the pools of 5 and 8 samples was similar to the individual sample test than in the pools of 10, hence it was decided to continue with the pool of 5 samples to validate the pooling method. A total of 470 unselected samples collected from individuals examined at the PGDL were tested individually and in pools of 5 (**S1 Fig**) for SARS-CoV-2 detection using the BGI and the Sansure-Biotech methods.

### Data analysis

The cycle threshold (Ct) values for individual (retest) and pooled samples were presented with mean ± standard errors. The Fisher’s exact test and the Mann-Whitney test were used to compare the test accuracy and the Ct values between both kits and between individual sample testing and the pooling method. The effect of pooling on the Ct values of the positive samples was evaluated using the Pearson’s correlation test. All data were analyzed using the R statistical software of version 4.0.3. A *p-*value < 5% was considered significant.

## Results

### Results of the individual retest

Of the 14 positive samples initially selected for the experimental pools, SARS-CoV-2 was confirmed in the retest for the eight samples pool using the BGI assay. For the Sansure-Biotech assay, SARS-CoV-2 was confirmed for seven samples pool in the retest. No statistically significant difference (Chi squared, *p*>0.05) was recorded between the two methods. A 92.86% (13/14) similarity was recorded for individual sample test result using the BGI and the Sansure-Biotech assays. Seven samples had a similar positive result for both kits, but six samples were negative for both kits. One of the samples (200902P3300403) had a late amplification (Ct = 37.17) detected with the BGI kit whereas no amplification was detected using the Sansure-Biotech kit.

### Test results for individual (retest) and pooled methods

The results of individual (retest) and experimental pools of 5, 8 and 10 are presented in **Table 2**. Comparing the results of the BGI method for the individual and pool of 5, a 100% similarity was recorded for the two methods. The sensitivity of the pool of 5 while keeping individual retest as gold standard was 100% (95% CI 59.04–100%) and specificity of 100% (95% CI 57.07–100%). For the pool of 8, a 100% test match with the individual retests resulted in sensitivity of 100% (95% CI 63.06–100%) and specificity of 100% (95% CI 57.07–100%). For the pool of 10, result match for 12 samples with the individual test giving a sensitivity of 75% (95% CI 34.91–96.81%) and a specificity of 100% (95% CI 57.07–100%). There was no significant difference (*p*>0.05) between the individual and pooling tests respectively. Using the Sansure-Biotech method, for each pool size, the results match for 100% with the individual test (retests). The sensitivity was 100% (95% CI 59.04–100%) for all the pool sizes tested with a specificity of 100% (95% CI 59.04–100%) keeping individual retest as gold standard with no statistically significant difference (*p*>0.05) between the individual and pooled sample tests.

**Table 2:** Results of the individual (retest) and experimental pooling method. Sensitivity and specificity of pool test are indicated by considering the individual test as gold standard.

	BGI				Sansure-Biotech			
Sample code	Individual (retest)	Pool of 5	Pool of 8	Pool of 10	Individual	Pool of 5	Pool of 8	Pool of 10
200831P9400167*	+	+	+	+	+	+	+	+
200903P8600016*	+	+	+	+	+	+	+	+
200902C2800011*	+	+	+	+	+	+	+	+
200921P8600060*	+	+	+	+	+	+	+	+
200923P0800072*	+	+	+	+	+	+	+	+
200921P9400141	+	+	+	+	+	+	+	+
200902P9400076	+	+	+	-	+	+	+	+
200902P3300403	+	*ER*	+	-	-	-	-	-
200901P6500072	-	-	-	-	-	-	-	-
200831P3300488	-	-	-	-	-	-	-	-
200925P3300629	-	-	-	-	-	-	-	-
200924C2500010	-	-	-	-	-	-	-	-
200921P3300452	-	-	-	-	-	-	-	-
200925P9501066	-	-	-	-	-	-	-	-
Total positive	8	7	8	6	7	7	7	7
Total negative	6	6	6	8	7	7	7	7
Invalid result	0	1	0	0	0	0	0	0
Sensitivity	-	100%	100%	75%	-	100%	100%	100%
Specificity	-	100%	100%	100%	-	100%	100%	100%

*: High viral load.

**+**: Positive result.

**-**: Negative result.

ER = Equivocal result.

### Cycle threshold (Ct) values of individual and pooled samples

**Fig 2:**
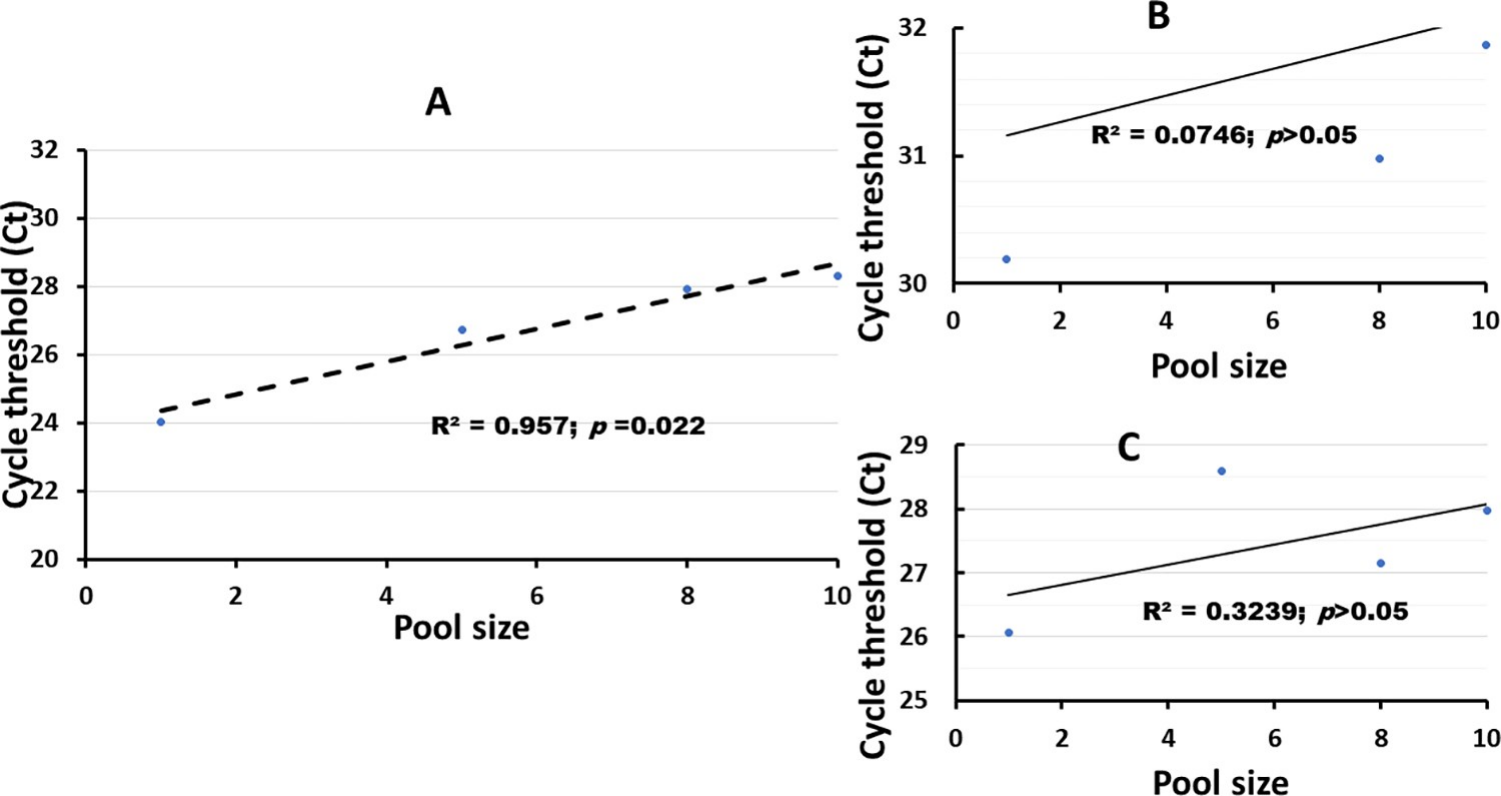
Correlation between the number of pooled samples and the cycle threshold (Ct) values. A. The BGI kit. B. The *ORF1ab* gene of Sansure-Biotech kit. C. The *N* gene of Sansure-Biotech kit. The analysis concerns only the five samples with high viral load.

Using the BGI assay, the cycle threshold (Ct) values of the *ORF1ab* gene for individual test differed within the range of 21.46 to 37.17 (**Table 3**). Whereas the Ct of pooled samples differed within the range of 25 to 33.17; 25.89 to 36.72 and 26.47 to 35.94 for the pool of 5, 8 and 10 respectively. The number of samples pooled was positively and significantly correlated with increasing Ct (Pearsons r = 0.96; *p* = 0.022, considering the 5 specimens with high viral load) (**Fig 2**). There was no significant difference in the mean Ct of the five positive samples between the individual test and pool of 5. However, a significant difference was observed between the individual test and pool of 8 (U = .2; *p* = 0.033) and between the individual test and pool of 10 (U = .2; *p* = 0.033).

**Table 3:** Range of cycle threshold (Ct) values of individual (retest) and pooled samples using BGI and Sansure-Biotech methods.

		BGI			Sansure-Biotech							
	*ORF1ab* (Ct)				*ORF1ab* (Ct)				N (Ct)			
Sample code	Ind	Pool of 5	Pool of 8	Pool of 10	Ind	Pool of 5	Pool of 8	Pool of 10	Indiv	Pool of 5	Pool of 8	Pool of 10
200831P9400167#	27.20	30.41	31.08	31.31	29.63	35.48	32.80	33.42	27.68	31.05	29.98	30.69
200903P8600016#	22.70	24.05	25.89	26.47	27.26	31.77	28.16	28.92	22.96	26.70	25.01	25.51
200902C2800011#	23.74	26.31	27.61	28.31	30.74	39.05	32.95	34.63	27.88	32.92	30.27	31.95
200921P8600060#	25.10	27.96	28.96	29.42	29.04	30.22	29.64	31.22	24.87	25.43	25.15	26.06
200923P0800072#	21.46	25.00	26.03	26.08	34.32	31.91	31.34	31.17	26.91	26.88	25.40	25.68
200921P9400141	28.42	33.20	34.07	35.94	--	--	--	--	35.59	34.03	34.53	33.93
200902P9400076	31.59	33.95	35.23	--	--	--		--	39.77	37.26	36.09	34.68
200902P3300403	37.17	IR	36.72	--	--	--	--	--	--	--	--	--
**Mean ± SD** *	24.04±1.1	26.74±1.3	27.91±1.1	28.32±1.1	30.19±1.3	33.68±1.8	30.98±1.0	31.87±1.1	26.06±1.1	28.59±1.6	27.16±1.4	27.97±1.5

**#:** Specimen with high viral load.

*: Only the 5 specimens with high viral load are include.

--: No amplification (negative).

IR: Invalid result (amplification failed).

Ind: Individual test.

Using the Sansure-Biotech method, the cycle threshold (Ct) values of the *ORF1ab* gene for the positive results in the individual retest differed within 27.26 and 34.32 (**Table 3**). Whereas they differed within the range of 30.22 to 39.05; 28.16 to 32.95 and 28.92 to 34.63 for the pools of 5, 8 and 10 samples respectively. A positive but non-significant correlation (Pearson’s *r* = 0.273; *p*>0.05) trend in the increase of Ct values with the pool size and no significant difference (Mann-Whitney, *p*>0.05) was recorded for all the pairwise comparisons of the mean Ct. The Ct values detected during individual retest for the *N* gene differed between 22.96 and 39.77, whereas they differed within the range of 25.43 to 37.26; 25.01 to 36.09 and 25.51 to 34.68 for the pools of 5, 8 and 10 samples respectively. Also, there was a positive and non-significant correlation (Pearson’s r = 0.569; *p*>0.05) between the Ct values and pool size for the five samples with high viral load.

A significant difference (U = 10.5; *p* = 0.04) in the mean Ct values for *ORF1ab* gene was observed between the individual samples tested using BGI (27.17±1.97) and those tested using the Sansure-Biotech (31.79±2.04).

### Validation of pooling results

Of the 470 samples tested, one was positive for SARS-CoV-2 using the individual RT-PCR test with overall prevalence of 0.21%. This positive sample had a Ct value of 32.97 (*ORF1ab* gene) using the BGI kit; 34.55 (*N* gene) and 37.86 (*ORF1ab* gene) for the Sansure-Biotech kit. Besides, out of the 94 pools of 5 created, one was SARS-CoV-2 positive and the positive pool was that with the positive sample as revealed by the individual testing regardless of the kit used. The Ct value of the positive pool was 33.48 (*ORF1ab* gene) using the BGI. Using the Sansure-Biotech, only the *N* gene was amplified with a Ct value of 36.23. For the 470 samples tested, the total number of RT-PCR reactions using the pooling method was 99 resulting in the conservation of 371 RT-PCR reactions for each kit used. The time used for testing was also divided by five using the procedure of pooled samples (**Table 4**).

**Table 4:** Comparative resource, time and result requirements between individual and pooled samples testing according to the BGI and Sansure-Biotech RT-PCR procedures.

		Number of samples	Number of RT-PCR	Time of manipulation (Mn)	Number of positive RT-PCR	Ct of the positive PCR	
						*ORF1ab*	*N*
Individual test	BGI	470	470	675	1	32.97	
	Sansure			500	1	37.86	34.55
Pooling test	BGI	470	99	270	1	33.48	
	Sansure			200	1	-	36.23

## Discussion

This study was designed to study the implementation of pooled samples for the detection of SARS-CoV-2 in Gabon using the BGI and Sansure-Biotech RT-PCR kits. The results obtained indicates the high sensitivity and specificity of the sample pooling procedure regardless of the kit used (BGI or Sansure-Biotech) compared to the individual test as gold standard.

Pooled samples testing requires highly sensitive RT-PCR kits to avoid missing low positive samples [15]. The BGI and Sansure-Biotech kits have a good performance as already reported [16] and the results of our study support the similarity of both. Indeed, individual testing of the 14 positive samples were similar for 13 of them for the BGI and Sansure-Biotech kits (92.86% similarity rate). Seven samples had a positive result match for the two kits, six had no detection for both kits and a mismatch result for one sample (at the limit of detection with BGI: Ct value 37.17 and no detection with Sansure-Biotech). All the positive samples confirmed in the individual retest by both kits were those with high viral load while samples with low viral load tested negative at retest. It is possible that the low load samples will become negative after long storage at -20°C. Indeed, the ideal temperature of -70°C for long term storage has been recommended to guarantee the non-degradation of RNA [5, 18]. The results mismatch (1/14) observed between the two kits could be explained by the detection limit which was 100 copies/mL for BGI (User manual page 4 of 8) and 200 copies/mL for Sansure-Biotech (Doc.#: 2019-nCoV Manual: V01).

For the maximum pool size that is the concern in the pooling method, the results of the present study show that with high viral load (Ct up to 27.20: **Table 3**), the test efficiency match was recorded between individual and all the pool sizes tested (maximum pool size tested in our study = 10) for both kits. This finding is similar to that of other studies showing that a single positive sample can be detected even in pools of up to 32 and 64 samples [15, 18]. However, for samples with low viral load, a result mismatch between individual and pooling methods was observed. Indeed, using the BGI kit, a single positive sample with Ct > 31 and up to 37.17 can be easily detected in up to 8 pooled samples. These results are similar to the finding that pool testing can facilitate the detection of a single positive sample with Ct value as high as 38 [19]. The detection of these samples (sample with Ct > 31 and up to 37.17) was impossible in a pool of 10. Samples with low viral load (with high Ct value) are often a concern for the accuracy of pool sample testing method [15]. It is advisable to re-amplify the RT-PCR products to reduce false negative results [18]. With the Sansure-Biotech assay no effect of pool size was observed in our study. For this kit, all samples with high viral load were easily detected for the two target genes in individual sample testing and in pools. However, for the samples with low viral load, only the target gene *N* was amplified for both the individual and pooling tests. The fact that it is a qualitative assay (it is positive for the presence of one of the two targeted genes) can explain this result. Our findings confirm the necessity to validate the pooling procedure for each kit considered [15].

An increase in the Ct values with pool size was recorded (**Fig 2**) in our study. These findings agree with that of other authors [8, 11, 18]. The increase in cycle threshold values in pooled samples indicate the need to use a method with high sensitivity because of a possible viral load dilution [20]. This pooling dilution effect may reduce the diagnostic accuracy of the kits used.

It was noticed for the Sansure-Biotech kit that the *N* gene was more sensitive than the *ORF1ab* (**Table 3**) and this observation agrees with the report of a study that established that the highly specific target gene (*ORF1ab*) is considered to be less sensitive than all the other target genes in the clinical diagnosis of SARS-CoV-2 [21]. Furthermore, in our study we noted that the Ct values of the *N* gene from Sansure-Biotech are closer to those of the *ORF1ab* gene (BGI) than those of the *ORF1ab* gene (Sansure-Biotech). In fact, the Ct values *ORF1ab* gene (Sansure-Biotech) are consistently higher than Ct values of *ORF1ab* gene (BGI) (**Table 3**). The difference in the RNA extraction procedure, in the primers and in the amplification efficiency could account for the difference in Ct values between the two tests.

The pooling method is being implemented in the PDGL using a two-stage approach consisting of one-time pooling followed by individual tests on positive pools. In the present study, the testing of 470 samples both individually and by pooling showed the same diagnostic accuracy regardless of the type of kit used (BGI or Sansure-Biotech). However, the pooled sample diagnostic strategy permitted to economize test resources (470 PCR for the individual tests vs 99 PCR for the pooling tests) and working time (mean of 600 minutes for the individual tests vs 200 minutes for the pooling tests): (**Table 4**). The diagnostic accuracy and duration of the test are fundamental elements for selecting the SARS-CoV-2 diagnostic method to recommend to laboratories involved in the COVID-19 response [15, 22]. Although the pooling methods has some advantages as mentioned, there also have some limitations for routine Covid-19 testing including: (i) it could delay the time frame for delivering results to the patients received first, (ii) an individual retesting is required to confirm the result and (iii) the pooled sampling method is not efficient during periods of high prevalence. Indeed, in our study the demonstration of test resource conservation by the pooling approach has been shown for prevalence of 0.002% (1/470) while the study of Shani-Narskiss et al. [17] showed that number of tests needed increase with prevalence. Thereby, in a higher prevalence situation (30% and above), the saving of reagents and time of the pooling over the individual testing may not as significant [23].

## Conclusions

The different methods used for the detection of SARS-CoV-2 needs to be evaluated and adapted according to the local situation for the control of the COVID-19 pandemic. From the present study, pooled samples testing is an interesting method with several challenges surrounding it. Indeed, the question ‘could pooled samples analysis affect results using BGI and Sansure-Biotcech RT-PCR assays used in Gabon for SARS-CoV-2 detection?’ can be answered by simply stating that it could be implemented in Gabon because it permits the laboratory to improve its testing capacity, economize reagents and time without affecting the diagnostic accuracy.

## Supporting information

S1 Fig Schematic view showing the basic structure of samples when tested by the individual and pooling methods in deep-well plates. A. 470 samples tested by individual in 5 different deep-well plates. B. 470 samples tested by pools of 5 in 1 deep-well plates. (BC and PC indicate the “blank control” and the “positive control” respectively.
(JPG)
