# Supplementary figures and images for: Could pooled samples method affect SARS-CoV-2 diagnosis accuracy using BGI and Sansure-Biotech RT-PCR kits used in Gabon, Central Africa?
Source: PLoS One. 2022 Jan 21;17(1):e0262733. doi: 10.1371/journal.pone.0262733 (PMC8782308; doi:10.1371/journal.pone.0262733)

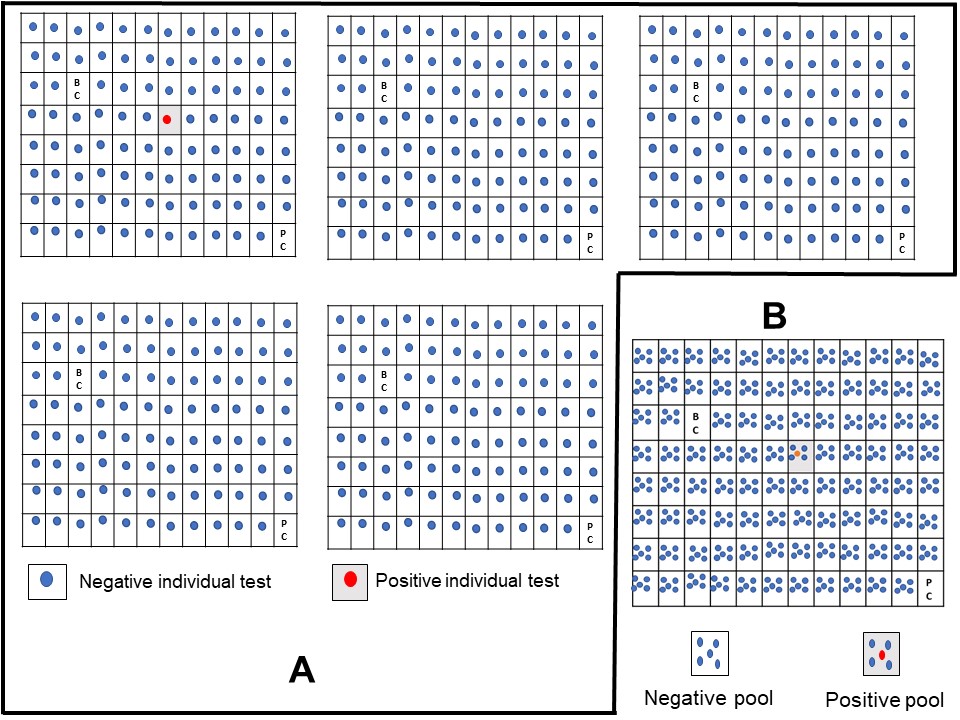

Supplement: S1 Fig — A. 470 samples tested by individual in 5 different deep-well plates. B. 470 samples tested by pools of 5 in 1 deep-well plates. (BC and PC indicate the “blank control” and the “positive control” respectively. (JPG) [file pone.0262733.s001.jpg]
